# The prognostic effect of immunosuppressive therapy in IgA nephropathy with stage 3 or 4 chronic kidney disease

**DOI:** 10.1080/0886022X.2021.1956536

**Published:** 2021-08-10

**Authors:** Xiaoxia Yang, Feng Ma, Ming Bai, Yan Wang, Qing Jia, Ruijuan Dong, Chunmei Liu, Shiren Sun

**Affiliations:** Department of Nephrology, Xijing Hospital, the Fourth Military Medical University, Xi’an, China

**Keywords:** IgA nephropathy, stage 3 or 4 chronic kidney disease, immunosuppressive, survival analysis, prognostic

## Abstract

**Background:**

It is debated whether patients with IgAN with heavy proteinuria and decreased eGFR benefit from aggressive treatment consisting of corticosteroids alone or combined with immunosuppressive agents.

**Methods:**

A retrospective study was performed between January 2008 and December 2016 on patients with IgAN who had urinary protein excretion > 1.0 g/d and an eGFR between 15 and 59 mL/min/1.73 m^2^. These patients were assigned to receive supportive care alone or supportive care plus immunosuppressive therapy. The primary outcome was defined as the first occurrence of a 50% decrease in eGFR or the development of ESKD.

**Results:**

All 208 included patients were followed for a median of 43 months, and 92 (44%) patients experienced the primary outcome. Cumulative kidney survival was better in the immunosuppression group than in the supportive care group (*p* < .001). The median annual rate of eGFR decline in the immunosuppression group was −2.0 (−7.3 to 4.2), compared with −8.4 (–18.9 to −4.1) mL/min/1.73 m^2^ in the supportive care group (*p* < .001). In multivariate Cox regression analyses, immunosuppressive therapy was associated with a lower risk of progression to ESKD, independent of age, sex, eGFR, proteinuria, MAP, kidney histologic findings and the use of RASi agents (HR = 0.335; 95% CI 0.209–0.601). Among the adverse events, infection requiring hospitalization occurred at similar rates in both groups (*p* = .471).

**Conclusion:**

Immunosuppressive therapy attenuated the rate of eGFR decline and was associated with a favorable kidney outcome in IgAN patients with heavy proteinuria and decreased eGFR, and the side effects were tolerable.

## Introduction

IgAN is recognized as the world’s most common primary glomerular disease with variable clinical presentation and progression rates that are dependent on the clinicopathological phenotype and duration of follow-up. Overall 4–40% of patients progress to end-stage kidney disease (ESKD) by 10 years [1]. For patients with IgAN at risk of progressive disease (those with proteinuria above 1.0 g/d, hypertension, or decreased eGFR [2,3], a number of supportive measures are recommended as a general approach for these patients, such as strict blood pressure (BP) control and the use of renin-angiotensin system inhibitor (RASi) agents [4]. These supportive measures are relatively safe but invariably not sufficient to prevent the progression of ESKD [5]. When the serum creatinine exceeds 3 mg/dL, 80% of patients do not receive RASi agents because of the associated adverse events [6]. Therefore, the selection of only those who would benefit from additional conservative management to strike the optimal balance between risk and benefit remains a challenge to the clinician, especially among patients with heavy proteinuria and decreased eGFR.

IgAN has characteristics of immune complex-mediated glomerulonephritis, and the dominant deposition of IgA in the mesangial area has been suggested as a critical factor in the pathogenesis of IgAN [7]. Corticosteroids and a series of immunosuppressants have been proposed to slow down the progression of IgAN [8–12]. However, the benefit of immunosuppressive therapy in patients with heavy proteinuria and decreased eGFR is controversial [13]. Corticosteroid therapy has demonstrated efficacy in reducing persistent proteinuria and preventing the progression of ESKD in a number of validation studies that only included patients with preserved kidney function [9,14]. Patients who have impaired kidney function have fewer treatment options. Beyond corticosteroids, a few studies have shown that immunosuppressive combination strategies may prevent ESKD progression. Nevertheless, these studies had a relatively small sample size, no control arm, a large dose of corticosteroids and a long course of treatment [8,10,15] and had similar entry criteria, which excluded patients with an eGFR of <30 mL/min/1.73 m^2^ at the time of biopsy and/or proteinuria >3.5 g/d [16]. On account of the insufficient treatment evidence and uncertainty of risk and benefit, the prognostic effectiveness of immunosuppressive combination strategies is still in question.

This retrospective study was performed to evaluate the effectiveness and safety of supportive care and immunosuppressive combination strategies in IgAN patients with proteinuria >1.0 g and stage 3 or 4 chronic kidney disease. We estimated the risk of progression based on the 50% decline in eGFR and the development of ESKD.

## Methods

### Study population

This study was a retrospective cohort study that was carried out at Xijing Hospital. We collected all kidney biopsy-proven IgAN patients between January 2008 and December 2016 in the Department of Nephrology. The indications for kidney biopsy were proteinuria >0.5 g, decreased eGFR, newly emerging hypertension, and/or hematuria, and the clinical experience of nephrologists. The inclusion criteria were as follows: IgAN proven by kidney biopsy, age between 16 and 80 years old, urinary protein excretion >1.0 g/d, impaired kidney function (defined as an eGFR between 15 and 59 mL/min/1.73 m^2^), and a follow-up time of 6 months or more. The exclusion criteria were as follows: systemic disease known to be associated with secondary IgAN (Henoch–Schönlein purpura, systemic lupus erythematosus, liver cirrhosis, etc.), crescent IgAN (defined as >50% crescentic glomeruli on kidney biopsy), minimal change disease with IgA mesangial area deposits, acute interstitial nephritis, acute kidney injury, or any prior treatment with immunosuppressants. The study protocol was approved by the Ethics Committee of our hospital (ethical number KY20213027-1).

### Study procedure and data collection

**Figure 1.**
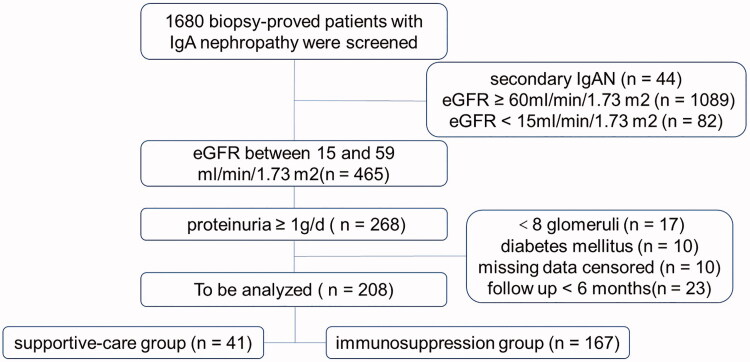
Flow diagram for inclusion of patients.

Originally, 1680 biopsy-confirmed IgAN patients were screened in our study. Finally, 208 IgAN patients who met the inclusion criteria were included (Figure 1). Among all collected kidney biopsy-confirmed IgAN patients, 57% received RASi agents, and 80% (*n* = 167) had immunosuppression therapy. Immunosuppressive regimens included corticosteroids (CS, 97%), cyclophosphamide (CTX, 67%, with pulse CTX in 38% and oral CTX in 62%), mycophenolate mofetil (17%), and calcineurin inhibitor (3%). The corticosteroid monotherapy group consisted of patients who received oral prednisone at a dose of 0.6–1.0 mg/kg/day for 2 months with subsequent gradual reduction (5 mg/day per month, tapered to 10 mg for 6 months). The low-dose CS + immunosuppressant therapy group consisted of patients who received oral prednisone at a dose of 0.4–0.6 mg/kg/day for 2–3 months with subsequent gradual reduction (5 mg/day per month, tapered to 10 mg for 6–12 months). Oral CTX was prescribed at 50 mg/day for 5 months, and pulse CTX was prescribed at 0.8 g per month for 6 months. The starting dose of MMF was 1–1.5 g/d for 6 months and then dosed by half for 6 months. Immunosuppressants other than CS were used in combination with CS as a first intention in 87% of patients. Otherwise, they were added after an initial course of CS.

Baseline demographic and clinical data were collected from electronic medical records at the time of the biopsy or the time closest to the patient's biopsy. Age, sex, and systolic and diastolic blood pressure were recorded. All follow-up data were updated to January 2019, and the results of laboratory tests of serum creatinine and 24 h urinary protein levels were also recorded. Urine protein excretion was quantified every 3 months until month 12 after the kidney biopsy, and every 6–12 months until the last follow-up. The eGFR was calculated using the Chronic Kidney Disease Epidemiology Collaboration equation [17]. Pathological indicators were reevaluated by one pathology expert who was blinded to the kidney outcomes and treatment protocols and was graded using the Oxford MEST-C scoring system [18]. We also investigated adverse events, including osteonecrosis of the femoral head, increased liver enzymes (alanine transaminase >80 IU/mL), newly diagnosed diabetes, leukopenia (leukocyte count <4000/μL) and infection requiring hospitalization during follow-up.

### Definitions

The primary outcome was kidney survival rate, defined as the first occurrence of a 50% decrease in eGFR, the development of ESKD (defined as an eGFR < 15 mL/min/1.73 m^2^, or initiation of chronic sustained dialysis for three months), or death due to kidney disease. The secondary outcomes included proteinuria changes evaluated by time-averaged proteinuria (calculated using the average of proteinuria measurements during the follow-up period in both groups), the rate of kidney function decline (slope of eGFR), or death due to any cause across the study visits.

### Statistical analysis

Data are presented using descriptive statistics such as the mean ± standard deviation, medians and interquartile ranges, and numbers with proportions. Intergroup differences were compared using an independent t test, nonparametric tests as appropriate, and Pearson’s chi-squared test for categorical variables. Kaplan–Meier curves were used to estimate cumulative kidney survival, and the log-rank test was used to compare between groups. Univariate and multivariable-adjusted Cox proportional hazards analyses were carried out to estimate which variables were associated with kidney survival. Variables were brought into a multivariable-adjusted Cox proportional hazards model using an enter method, and the hazard ratio (HR) resulted from adjusted models: model 1 was adjusted for demographic and clinical data; model 2 was adjusted for the variables in model 1 plus RASi; model 3 was adjusted for the variables in model 1 plus histological data; and model 4 was adjusted for the variables in model 3 plus RASi. The results are presented as hazard ratios (HRs) and 95% confidence intervals (CIs). Statistical significance was defined as a two-sided *p* value < .05. The above statistical analyses were performed by using SPSS statistics software 17.0 for Windows (IBM Corporation, Armonk, NY).

## Results

### Patients’ baseline clinical and pathological characteristics

The baseline clinical and pathological characteristics of the included patients are shown in Table 1. A total of 208 eligible IgAN patients with urinary protein > 1.0 g/d and decreased eGFR were enrolled and monitored in this study. The proportion of patients who were male was higher in the immunosuppression group (*p* = .004), but age, systolic, diastolic, and mean arterial pressure (MAP) did not differ between groups at the time of kidney biopsy (*p* > .05). The mean eGFR was 39.4 ± 12.0 mL/min/1.73 m^2^ in the immunosuppression group and 39.4 ± 14.4 mL/min/1.73 m^2^ in the supportive care group (*p* = .188). Likewise, the mean urinary protein excretion did not differ between the two groups, as it was 2.8 ± 2.4 g in the immunosuppressive therapy group and 2.5 ± 1.8 g in the control group (*p* = .992).

**Table 1.** Comparison of baseline clinical features between different Therapeutic Schedule.

Characteristic	supportive group (*n* = 41)	immunosuppression group (*n* = 167)	*p* value
Baseline (kidney biopsy)			
Sex (male/female)	32/9	88/79	.004
Age, years	35.5 ± 12.2	37.7 ± 13.0	.333
Blood pressure (mmHg)			
Systolic	145.7 ± 18.6	143.3 ± 23.1	.542
Diastolic	94.4 ± 15.0	90.9 ± 15.2	.188
MAP	111.5 ± 15.3	108.4 ± 16.7	.278
Serum creatinine (umol/L)	203.7 ± 84.4	187.9 ± 64.1	.188
eGFR (mL/min/1.73 m2)	39.4 ± 14.4	39.4 ± 12.0	.992
CKD stage			
Stage 3	29(71)	123 (74)	
Stage 4	12(29)	44 (26)	
Proteinuria, g/24h	2.5 ± 1.8	2.8 ± 2.4	.339
1–3.5g	33 (80)	131 (78)	.774
≥3.5g	8 (20)	36 (22)	.774
Oxford histological score			
M1	20 (49)	46 (28)	.014
E1	3 (7)	43 (26)	.011
S1	23 (56)	95 (57)	.927
T0	15 (37)	66 (40)	.858
T1	11 (26)	59 (35)	.359
T2	15 (37)	42 (25)	.171
C0	28 (68)	72 (43)	.005
C1	10 (25)	71 (43)	.048
C2	3 (7)	24 (14)	.304
Treatment			
RASi under follow-up	17 (42)	100 (60)	.037

Values for categorical variables were given as count (percentage); values for continuous variables, as mean ± standard. Abbreviation: eGFR, estimated glomerular filtration rate, MAP, mean arterial pressure; RASi, renin angiotensin system inhibitor; Oxford histological score, M1 indicates mesangial score >0.5; E1, endocapillary hypercellularity (any glomeruli); S1, segmental glomerulosclerosis (any glomeruli); T0, tubular atrophy/interstitial fibrosis (<25% of cortical area); T1, tubular atrophy/interstitial fibrosis (25%–50% of cortical area); T2, tubular atrophy/interstitial fibrosis (>50% of cortical area); C0, no crescents; C1, crescents (<one-fourth of glomeruli); C2, crescents (>one-fourth of glomeruli).

The pathological data are also listed in Table 1. Compared to the supportive care group, there were more patients with M and E lesions in the immunosuppression group. Crescents were present in 57% of the immunosuppressive therapy group and 32% of the supportive care control group (*p* = .005, Table 1).

### Kidney survival from baseline

**Figure 2.**
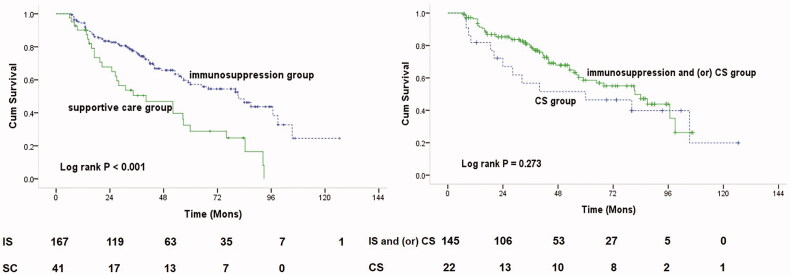
Kaplan–Meier plots of renal survival curves for primary outcome. (a) Renal survival according to patients with immune-suppression group (IS) versus supportive care (SC) group. (b) Renal survival according to patients with IS and (or) corticosteroids (CS) versus CS group.

A total of 208 patients with heavy proteinuria and chronic kidney disease stages 3 through 4 in IgAN were followed for a median period of 42.6 ± 27.5 months; 92 (44%) progressed to the primary outcome. As shown in Figure 2, K-M survival analysis showed that 68% (28 out of 41) and 38% of (64 out of 167) patients reached the combined outcome in the supportive care and immunosuppression groups, respectively (*p* = .001). The cumulative kidney survival rate from baseline was better in the immunosuppression therapy group than in the control group (*p* < .001, Figure 2a). However, the cumulative survival rate was not significantly different in the CS group compared with the rate in the CS and/or immunosuppression group (*p* = .273, Figure 2b). The 5- and 8-year kidney survival rates were 57% and 37% in the immunosuppression therapy group, and 29% and 0% in the control group, respectively. Patients who received immunosuppressive therapy had a lower risk of composite outcomes, including ESKD, kidney replacement therapy, and a 50% reduction in eGFR (*p* < .05, Table 2). The mean annual rate of eGFR decline in the immunosuppression group was −2.0 (–7.3 to 4.2) mL/min/1.73 m^2^, compared with −8.4 (–18.9 to −4.1) mL/min/1.73 m^2^ in the supportive care group (*p* < .001, Table 2). Analyses using annual changes in the inverse of serum creatinine level instead of eGFR showed similar results. Only 1 patient died due to any cause, with no clear difference between the two groups.

**Table 2.** End points on the basis of the available patients at the end of the study phase.

End point	supportive group (*n* = 41)	immunosuppression group (*n* = 167)	*p* value
Follow-up parameters and outcomes			
Mean follow-up time (month)	36.9 ± 27.0	44.0 ± 27.6	.142
eGFR (mL/min/1.73 m2)	17.2 ± 18.4	38.1 ± 29.1	<.001
Primary outcomes, No. (%)	28 (68)	64 (38)	.001
eGFR decreas*e* ⩾50%	28 (68)	47 (28)	<.001
Onset of ESKD	27 (66)	50(30)	<.001
Kidney replacement therapy	20 (49)	48 (29)	.005
Death due to kidney disease	0 (0)	0 (0)	–
Secondary outcomes			
Death due to any cause	0 (0)	1(0.9)	.803
Time-averaged proteinuria, g/d	1.9 ± 1.0	1.7 ± 1.5	.335
Rate of kidney function decline, mL/min/1.73 m2 per year	–8.4 (–18.9 to −4.1)	–2.0 (–7.3 to 4.2)	<.001

*p* value for primary outcome calculated using survival analysis. Other *p* values calculated using *t*-tests or Fisher exact tests. Rate of kidney function decline defined for each individual patient using the slope from least-squares linear regression of all eGFR estimates over time. Abbreviations: ESKD, end-stage kidney disease.

### Risk factors for the primary outcome in the study population

The HR for kidney survival was assessed by Cox -proportional hazards analysis (Table 3). Univariate analysis showed that immunosuppressive therapy improved the chance of kidney survival (HR = 0.439, 95% CI 0.280–0.687, *p* < .001); multivariate analysis adjusted for the demographic and clinical factors of sex, age, mean arterial pressure, proteinuria, and eGFR showed that immunosuppressive therapy was significantly associated with a decreased risk of kidney outcome (HR = 0.375, 95% CI 0.235 − 0.599, *p* < .001) (Table 3, Model 1). In addition, when adjusted for the use of RASi agents or histological parameters, the studies yielded similar results, indicating a protective kidney function effect from immunosuppressive therapy (Table 3, Model 2 and 3). Furthermore, in the fully adjusted model, which included the histological parameters and the use of RASi agents, there was a persistent and significantly decreased risk of adverse outcomes as a result of immunosuppressive therapy (HR = 0.335, 95% CI 0.209 − 0.601, *p* < .001) (Table 3, Model 4).

**Table 3.** Effects of immunosuppressive therapy on kidney survival IgA nephropathy patients with decreased eGFR.

Immunosuppressive therapy	HR (95% CI)	*p* value
Univariate	0.439 (0.280–0.687)	<.001
Multivariate model 1^a^	0.375 (0.235–0.599)	<.001
Multivariate model 2^b^	0.427 (0.265–0.687)	<.001
Multivariate model 3^c^	0.334 (0.198–0.564)	<.001
Multivariate model 4^d^	0.335 (0.209–0.601)	<.001

^a^Model 1 was adjusted for age, sex, MAP, proteinuria, and eGFR.

^b^Model 2 was adjusted for age, sex, MAP, proteinuria, eGFR, and RASi.

^c^Model 3 was adjusted for age, sex, MAP, proteinuria, eGFR, M1, E1, S1, T1-2, and C1-2.

^d^Model 4 was adjusted for age, sex, MAP, proteinuria, eGFR, M1, E1, S1, T1-2, C1-2, and RASi.

A prespecified subgroup analysis showed evidence of significant protective effects of immunosuppressive therapy on the composite renal failure outcome in subgroups defined by baseline proteinuria (<3.0 and ≥3.0 g/d), age (<60 y), sex (male), kidney function (eGFR ≥30 mL/min/1.73 m^2^) and the use of RASi agents (Table 4). No significant protective effects were found in age (>60 y), sex (female), kidney function (eGFR <30 mL/min/1.73 m^2^) or the nonuse of RASi agents (Table 4) but were limited by the small sample size and primary outcomes.

**Table 4.** Prespecified subgroup analysis of the primary composite outcome.

Characteristic	HR (95% CI)	*p* value
Baseline age		
> =60y (*n* = 12)	0.552 (0.050–6.118)	.628
<60y (*n* = 196)	0.440 (0.277–0.699)	.010
Sex		
Male (*n* = 120)	0.367 (0.209–0.646)	.001
Female (*n* = 88)	0.628 (0.276–1.428)	.267
Baseline eGFR		
> =30 mL/min/1.73m^2^ (*n* = 152)	0.369 (0.205–0.663)	.001
<30 mL/min/1.73m^2^ (*n* = 56)	0.583 (0.287–1.186)	.137
Baseline proteinuria		
> =3 g/d (*n* = 53)	0.328 (0.141–0.746)	.008
< 3 g/d (*n* = 155)	0.482 (0.283–0.823)	.007
Use of RASi		
Yes (*n* = 117)	0.477(0.281–0.810)	.006
No (*n* = 91)	0.645(0.262–1.590)	.341

### Adverse events

Over the mean period of 43 months of follow-up, 12 patients (29%) with at least one first AE were observed in the supportive care group, and 59 patients (35%) were observed in the immunosuppression group (*p* = .582, Table 5). Among these adverse events, the number of hospitalizations required for infection was similar between the two study groups (*p* = .471). In the immunosuppression group, 12 patients were hospitalized with infections (ten with pneumonia and two with intestinal infection). One patient who received mycophenolate mofetil plus glucocorticoid therapy in the immunosuppression group died of sepsis. Three patients in the immunosuppression group underwent hemodialysis for acute-on-chronic kidney disease, while one patient was dependent on dialysis. Other patients did not receive kidney replacement therapy. We did not observe more hepatotoxic events, serious femoral head necrosis, new-onset diabetes mellitus, or leukopenia in the immunosuppression group than in the supportive care group.

**Table 5.** Adverse events during the follow-up period.

	Supportive group (*n* = 41)	immunosuppression group (*n* = 167)	*p* value
Total AEs	11 (27)	57 (34)	.372
Pneumonia	1 (2)	10 (6)	.696
intestinal infection	0 (0)	2 (1)	1.000
Osteonecrosis of the femoral head	0 (0)	0 (0)	–
Increase of liver enzymes (AL*T* > 80 IU/mL)	3 (7)	20 (12)	.579
Newly diagnosed diabetes	3 (7)	17 (10)	.771
leukopenia	4 (10)	8 (5)	.258

Multiple occurrences of the same AE in one person were only counted once. AE: adverse event; ALT: alanine aminotransferase; *p* value for comparisons between the number of patients in the supportive group and the number of patients in the immunosuppression group.

## Discussion

In this study, we retrospectively investigated the prognostic effect of immunosuppressive therapy in IgAN patients with heavy proteinuria and decreased eGFR. Compared with the supportive care control group, the cumulative kidney survival rate from baseline was significantly higher in the immunosuppressive therapy group. This protective value of immunosuppressive therapy was independent of baseline proteinuria and eGFR. In fact, the decline in eGFR was significantly slowed after initiation of immunosuppressive therapy. On the whole, our findings suggest that immunosuppressive therapy has advantages over supportive care therapeutic regimens in IgAN patients and tolerable low side events, at least in patients who have heavy proteinuria and a progressive decline in eGFR.

A number of clinical studies have demonstrated a positive effect of immunosuppressive combination therapy in IgAN [8,10,11]. For instance, in IgAN patients with decreased eGFR, Ballardie and Roberts [10] showed beneficial effects on kidney survival of a combined therapy of corticosteroids plus oral cyclophosphamide for 3 months and subsequent oral azathioprine for at least 2 years. Unfortunately, their study had a relatively small sample size (*N* = 38), and there was no control arm of patients not receiving immunosuppressive therapy in either study. A recently published retrospective analysis of a large European cohort VALIGA study [19] suggested particular kidney benefits from corticosteroids for patients with IgAN with a baseline eGFR below 50 mL/min/1.73 m^2^ as well as those with proteinuria above 3 g/d. In contrary, the STOP-IgAN [16] trial suggested that adding immunosuppression to intensive supportive care demonstrated no superiority in the improvement of the outcome. These obviously discrepant findings might be interpreted by a study of objective characteristics. Ballardie [10] and VALIGA [19] included patients with a low eGFR (< 50 mL/min/1.73 m^2^) and/or a rapidly progressive clinical course. Finally, the immunosuppressive regimen was strongly associated with the kidney survival rate in patients with a significant reduction in eGFR. Nevertheless, the STOP-IgAN study [16] excluded patients with high-grade proteinuria and moderate to severe decreased eGFR, and the eGFR decline was less than 2 mL/min/1.73 m^2^ per year before treatment in the intervention group. These results demonstrated that the superior benefits of immunosuppressive therapy on kidney survival might not be more remarkable than those of supportive care management in non-progressive IgAN. The rate of eGFR decrease among patients in the supportive care group of our study was higher than that in the STOP-IgAN study (−8.4 vs −1.6 mL/min/1.73 m^2^ per year). Possible reasons for the difference include the fact that participants in this trial were at higher risk progressively because of higher mean baseline proteinuria, and also because individuals of Eastern Asian (e.g., China, Philippines, Japan, and Vietnam) origin may have more rapid rates of eGFR decline and renal failure [20]. The annual rate of eGFR decline in STOP-IgAN was also lower than that observed in a cohort study [19] and a recent trial [21], both from Europe. The longer follow-up of the present trial compared with the STOP IgAN trial is another possible reason. Our study included patients with proteinuria > 1.0 g and chronic kidney disease stage 3 or 4, had a larger sample size and longer follow-up time, compared immunosuppressive therapy with optimal supportive care treatment, and found that immunosuppressive therapy attenuated the rate of kidney function decline, which may be an advantage over Ballardie et al. [10] and the VALIGA [19] study. In addition, our study showed that immunosuppressive therapy was consistently associated with a lower risk of adverse kidney disease outcome in multivariable analyses after adjustment for various clinical, histology and RASi factors. Consistent with our previous study [22], the overall 5-year survival rate in the immunosuppression group was 57%, which was more significant than the 29% in the supportive care group. Thus, this study might help create an effective immunosuppressive treatment regimen in improving kidney prognosis in patients with IgAN with heavy proteinuria and chronic kidney disease stages 3 through 4.

Moreover, subgroup analysis showed that compared with patients who had eGFR < 30 mL/min/1.73 m^2^, immunosuppressive therapy had protective effects on the composite renal failure outcome in the eGFR ≥ 30 mL/min/1.73 m^2^ subgroup, which may partly be due to poor prognosis of decreased eGFR. Likewise, subgroup analysis found that in patients who did not use RASi agents, immunosuppressants did not have more kidney protection advantages than the control group. These results might be explained by the worse kidney function in the group that did not use RASi agents (34 mL/min/1.73m2 in the nonuse of RASi group vs 43 mL/min/1.73m2 in the use of RASi group, *p* < .001), which was consistent with previous studies [16,23]. Hence, the use of immunotherapy in these patients requires a comprehensive assessment of efficacy and safety, and may be associated with relatively preserved kidney function, a lower dose of immunosuppressants, and a shorter treatment course.

Though an effective treatment regimen for patients with IgAN, immunosuppressants are associated with many adverse events, including inducing or aggravating infection, hepatotoxic events, metabolism disorders, and leukopenia [24]. Both immunosuppressive therapy and impaired kidney function can induce vulnerability to infection [16,25]. Therefore, it is worth discussing the dose of corticosteroids and immunosuppressants [26]. The TESTING [23] randomized clinical trial suggested that a full-dose corticosteroid regimen in patients with IgAN at high risk improved proteinuria and eGFR levels but found high rates of serious adverse outcomes. This conclusion might be related to the full-dose corticosteroid (0.6–0.8 mg/kg/d) regimen used in this study. In our study, during the follow-up period, a total of 12 patients (7%) suffered from infection requiring hospitalization, and only two patients (1%) suffered from serious femoral head necrosis during the observation period in the immunosuppression group. However, we observed that most infection sufferers had relatively mild symptoms, and only one death was recorded; other patients fully recovered after receiving appropriate therapy. This may be due to a lower dose of corticosteroids (30 mg/d) and immunosuppressants (maintenance dose) and the shorter course of (within 1 year) used in our study. Consistent with our study, a recent meta-analysis [11] suggested that small doses of immunosuppressants may reduce the incidence of adverse reactions, further illustrating that there was no significant difference in the incidence of adverse reactions between the immunosuppression and control groups. Because we only collected data on hospitalization for severe infections, the incidence of adverse reactions might be underestimated in our retrospective study. However, the incidence in our study is consistent with that in previous studies [16,26,27]. Overall, the side effects of immunosuppressive therapy are tolerable, and the regimen of immunosuppressive and combined treatment may provide a better option for IgAN patients with decreased eGFR.

Although our results are promising, several limitations should be acknowledged. First, this was a single-center retrospective observational study, and missing data were inevitable. There were some differences in histological backgrounds between the cohorts, although they were not significant after matching. Second, nothing is known about compliance with treatment, the occurrence of adverse events with either therapy, and the doses of corticosteroids and RASi agents; only 57% of patients received RASi agents in all patients. The use of RASi agents was not distributed evenly between the supportive care group and the immunosuppression group in the original cohort (42% verse 60%, *p* = .037). Third, due to the limitation of sample size, we did not further analyze the response of patients with eGFR < 30 mL/min/1.73 m^2^ to immunotherapy. Finally, we could not show the results of appropriate comparison between immunosuppression and supportive care because of lower number in the supportive care group and the strong bias resulting from the selection of therapeutic protocols at our institutions. Although ethnic differences in response to treatment and the longer follow-up, the rapid decline eGFR of supportive care group may still have a bias for the study’s results. Therefore, a large prospective cohort study should be performed to verify our findings.

## Conclusions

We showed that immunosuppressive therapy slowed the rate of kidney function decline and improved long-term kidney outcomes in IgAN patients with heavy proteinuria and decreased eGFR. Although the adverse events directly related to immunosuppressive therapy are tolerable, side effects still need to be closely monitored during medication. These findings have practical clinical implications in the treatment of IgAN patients with chronic kidney disease stage 3 or 4. Further multicenter clinical trials should be performed to verify our findings.
